# An Account of a Remarkable Spasmodic Affection

**Published:** 1784

**Authors:** 


*September* 16, 1784.

---

II. *An account of a remarkable ſpaſmodic affection. Communicated in two letters to Dr.* Simmons, *by*

*by Mr.* William Hulke, *Surgeon at Deal, in Kent.*

M. B. a maiden lady, aged 40, of a healthy complexion, in the beginning of the year 1782, began to be troubled with a ſingular complaint, which ſhe deſcribed in the following manner: Every night, ſoon after ſhe had fallen aſleep in bed, ſhe was ſuddenly awakened, ſhe ſaid, by a ſenſation of cold and numbneſs in the *outer ſide* of her right foot, which numbneſs gradually extended up her leg and thigh to her ſtomach, unleſs ſhe prevented it, by riſing from her bed inſtantly, and placing her foot on the floor; as ſhe always found that this gave her relief, and prevented the progreſs of the diſorder to her ſtomach. Suppoſing her complaint to be the cramp, ſhe took no notice of it, till after a fit of the gout, which attacked her in both feet, in March 1783, and laſted near three months, during which time ſhe paſſed her nights comfortably; but when the gouty paroxyſm went off, her former complaint returned with increaſed violence, and obliged her to have recourſe to medicine. All the remedies uſually given in nervous caſes were tried, and, among others (by the advice of of a neighbouring phyſician) the application of bliſters to the leg and foot, but without mitigating the complaint in the ſlighteſt degree. The numbneſs and ſtoppage of circulation (as ſhe expreſſes it) ſpread now with great quickneſs from the foot upwards, and, if not timely prevented from reaching the ſtomach, impede reſpiration, and bring on ſlight ſpaſms. To prevent or remove this degree of the paroxyſm, a perſon is conſtantly with her, that ſhe may, as ſoon as ſhe is attacked, be got out of bed, and place her foot on the floor, as this alone gives her immediate relief. She was once affected in the day-time, when ſhe had fallen aſleep in her chair. During each paroxyſm, ſhe ſweats violently; and when the fit has been ſevere, conſtantly throws up a quantity of taſteleſs, viſcid phlegm. Her menſes have hitherto been regular; her appetite is good, and till attacked with this diſorder, ſhe enjoyed an uninterrupted ſtate of good health.

*Deal,*

*Sept.* 16, 1784.

---

To the account of the caſe I communicated to you in September laſt, I now beg leave to add, add, that the patient, after trying a variety of remedies, without ſucceſs, has lately applied to an Empiric, who happened to be in this part of the world. He has given her ſome fœtid pills, which ſhe takes at night; an emetic mixture, which ſhe takes in the morning, and an ointment with which ſhe rubs her foot.—This method (whether from the force of imagination or not, I will not pretend to determine) has relieved her; her paroxyſms being now both more ſlight and leſs frequent than before.

